# Pridopidine Promotes Synaptogenesis and Reduces Spatial Memory Deficits in the Alzheimer’s Disease APP/PS1 Mouse Model

**DOI:** 10.1007/s13311-022-01280-1

**Published:** 2022-08-02

**Authors:** Héctor M. Estévez-Silva, Germán Cuesto, Ninovska Romero, José Miguel Brito-Armas, Abraham Acevedo-Arozena, Ángel Acebes, Daniel J. Marcellino

**Affiliations:** 1 Departamento de Ciencias Médicas Básicas, Instituto de Tecnologías Biomédicas (ITB), Universidad de La Laguna, Tenerife, Spain; 2 Department of Integrative Medical Biology, Umeå University, Umeå, Sweden; 3 Unidad de Investigación, Hospital Universitario de Canarias, ITB-ULL/CIBERNED, Tenerife, Spain

**Keywords:** Alzheimer’s disease, Neurodegeneration, PRE-084, ACR16, Sigma-1 receptor, Neuroprotection

## Abstract

Sigma-1 receptor agonists have recently gained a great deal of interest due to their anti-amnesic, neuroprotective, and neurorestorative properties. Compounds such as PRE-084 or pridopidine (ACR16) are being studied as a potential treatment against cognitive decline associated with neurodegenerative disease, also to include Alzheimer’s disease. Here, we performed in vitro experiments using primary neuronal cell cultures from rats to evaluate the abilities of ACR16 and PRE-084 to induce new synapses and spines formation, analyzing the expression of the possible genes and proteins involved. We additionally examined their neuroprotective properties against neuronal death mediated by oxidative stress and excitotoxicity. Both ACR16 and PRE-084 exhibited a concentration-dependent neuroprotective effect against NMDA- and H_2_O_2_-related toxicity, in addition to promoting the formation of new synapses and dendritic spines. However, only ACR16 generated dendritic spines involved in new synapse establishment, maintaining a more expanded activation of MAPK/ERK and PI3K/Akt signaling cascades. Consequently, ACR16 was also evaluated in vivo, and a dose of 1.5 mg/kg/day was administered intraperitoneally in APP/PS1 mice before performing the Morris water maze. ACR16 diminished the spatial learning and memory deficits observed in APP/PS1 transgenic mice via PI3K/Akt pathway activation. These data point to ACR16 as a pharmacological tool to prevent synapse loss and memory deficits associated with Alzheimer’s disease, due to its neuroprotective properties against oxidative stress and excitotoxicity, as well as the promotion of new synapses and spines through a mechanism that involves AKT and ERK signaling pathways.

**Supplementary Information:**

The online version contains supplementary material available at 10.1007/s13311-022-01280-1.

## Introduction

AD is a progressive neurodegenerative disease characterized by memory deficits and a gradual deterioration of cognitive functions [1]. Synaptic loss and the altered expression of synaptic and activity-dependent genes are early pathological events in the onset of AD and other diseases associated with dementia that precede extensive neuronal degeneration [2, 3]. Indeed, in Alzheimer’s patients, synapse dysfunction and loss, which occurs parallel with the formation of neurofibrillary tangles, correlate with cognitive impairment in contrast to other neuropathic features, such as amyloid plaques or neuronal death [3, 4].

Evidence suggests that alterations in sigma-1 receptor (σ1R) function are related to AD pathogenesis [5]. Furthermore, apolipoprotein E (ApoE), which is well known to be a genetic risk factor for AD, has been found to interact genetically with σ1R polymorphisms with a negative impact on the development of the disease [5]. The σ1R is a transmembrane protein widely expressed in the CNS and a resident protein of specific lipid raft-like regions of endoplasmic reticulum (ER) known as mitochondria-associated ER membranes (MAMs) [5]. Apart from its localization in MAMs, σ1Rs can act both as a chaperone and a co-receptor to other transmembrane proteins and modulate voltage-gated calcium-, potassium-, and sodium channels, NMDA receptors (NMDARs), and several G-protein coupled receptors [5]. Converging in vitro and in vivo data point to σ1R as a pharmacological target in neurological and psychiatric disorders [5].

Pridopidine (ACR16), although initially reported to act by antagonizing dopamine D_2_ receptors, [6, 7] was later described to display high affinity for σ1R in the nanomolar range and to preferentially occupy σ1R in vivo at behaviorally relevant doses [8, 9]. To date, ACR16 has been extensively evaluated preclinically and in clinical trials as a potential therapeutic agent to alleviate motor symptoms associated with Huntington’s disease (HD) [5], albeit its efficacy in HD requires further evaluation [10]. Nevertheless, accumulating evidence points towards the neuroprotective properties of ACR16 related to its specific binding to σ1R [11, 12]. In turn, 2-(4-morpholinethyl)1-phenylcyclohexanecarboxylate (PRE-084) is a phencyclidine (PCP) derived compound that results in a new highly selective agonist for σ1R that exhibited a low affinity for PCP binding sites [13]. Several published works emphasize the anti-amnesic, neuroprotective, and neurorestorative properties of PRE-084 [13].

In the present study, the neuroprotective and spinogenic effects produced by ACR16 and PRE-084 were evaluated in vitro using primary rat hippocampal cultures. Moreover, since only ACR16 generated dendritic spines involved in new synapses formation, we performed in vivo studies using ACR16 in the APP/PS1 mouse model for AD. This model exhibits an early loss of dendritic spines [14, 15] that might be correlated with impaired cognition [16, 17].

## Methods

### Reagents

ACR16 hydrochloride was provided from Axon Medchem, (Groningen, NL) and PRE-084 hydrochloride from Tocris (Bristol, UK). Synapsin1 protein was detected using either rabbit anti-synapsin1 polyclonal antibody or rabbit anti-synapsin1 (D12G5) XP monoclonal antibody (Catalog. #2312 and #5297, respectively; Cell Signaling Technologies, Danvers, MA). Antibodies against spinophilin (E1E7R) (rabbit monoclonal, Catalog. #14,136, Cell Signaling Technologies, Cambridge, UK), microtubule-associated protein 2 (MAP2B) (rabbit polyclonal, Catalog. #4542, Cell Signaling Technologies), EGFP (rabbit polyclonal, Catalog. #SK201, DAKO, Glostrup, DK), VGLUT1 (polyclonal guinea pig, Catalog. #AB5905, Merck-Millipore, Burlington, MA), Phospho-Akt (Thr308) (rabbit polyclonal, Catalog. #9275, Cell Signaling Technologies), pan Akt (C67E7) (rabbit monoclonal, Catalog. #4691, Cell Signaling Technologies), α-actin (mouse monoclonal, Catalog. #A2547, Sigma-Aldrich, St. Louis, MO), α-GFP (rabbit polyclonal, # A-11122, Invitrogen), total ERK-1/2 (mouse monoclonal, Catalog. #M8159, Sigma-Aldrich), and Phospho-ERK 1/2 (mouse monoclonal, Catalog. #M8158, Sigma-Aldrich) were also used. Polyclonal goat anti-mouse immunoglobulins/HRP (Catalog. #P0447, DAKO), goat anti-rabbit immunoglobulins/HRP (Catalog. #Ab6721, Abcam), goat anti-mouse IgG-Alexa Fluor 594 (Catalog. #A11005), goat anti-rabbit IgG-Alexa Fluor 488 (Catalog. #A11008) and 555 (Catalog. #A21428), and goat anti-guinea pig IgG-Alexa Fluor 488 (Catalog. #A11073) (Thermo Fisher Scientific, Waltham, MA, USA) were used as secondary antibodies.

### Primary Cell Cultures

Mixed primary cell cultures, hippocampal neurons, and astrocytes were prepared from Sprague–Dawley rat pups at P0, following protocols published in Banker and Goslin [18] and Morales et al. [19]. Hippocampal neurons were seeded at 1.5 × 10^5^ cells per cm^2^ onto #1 glass coverslips (Thermo Fisher Scientific, Waltham, MA, USA) and cultured as we have previously described [20, 21].

### Transfection

For distinguishing the dendritic arborization of individual neurons, cell suspensions were transfected to express EGFP before plating. To this end, we employed EGFP construct based on the GFP protein fused to chick β-actin under the regulation of the platelet-derived growth factor (PDGF) [19, 22] that was a gift from Yukiko Goda. Electroporation was performed using the kit from AMAXA (AMAXA Nucleofector kit; Lonza, Basel, Switzerland) and Nucleofector Core Unit (Lonza, Basel, Switzerland) as previously described [20].

### Neuronal Toxicity and Survival

Two different methods of neuronal toxicity were studied: excitotoxicity mediated by N-methyl-D-aspartate receptor (NMDAR) [23] and oxidative damage generated by hydrogen peroxide (H_2_O_2_) [24] in 12 DIV primary hippocampal cultures. We then evaluated the extent of toxicity (or neuroprotection) 24 h after insult. Two different concentrations of each σ1R ligand were evaluated, an intermediate concentration and a low concentration, added 15 min prior to the addition of NMDA or H_2_O_2_. The concentrations chosen were aimed to ensure ligand-receptor specificity and were determined by inhibition constants (K_i_) for ACR16 and PRE-084 determined by others and our own previous studies [8, 9, 25]. A low concentration was defined as the K_i_ value at σ1R (70 nM, ACR16 and 50 nM, PRE-084) while an intermediate concentration was 10 times higher than their respective K_i_ value at σ1R.

**Fig. 1:**
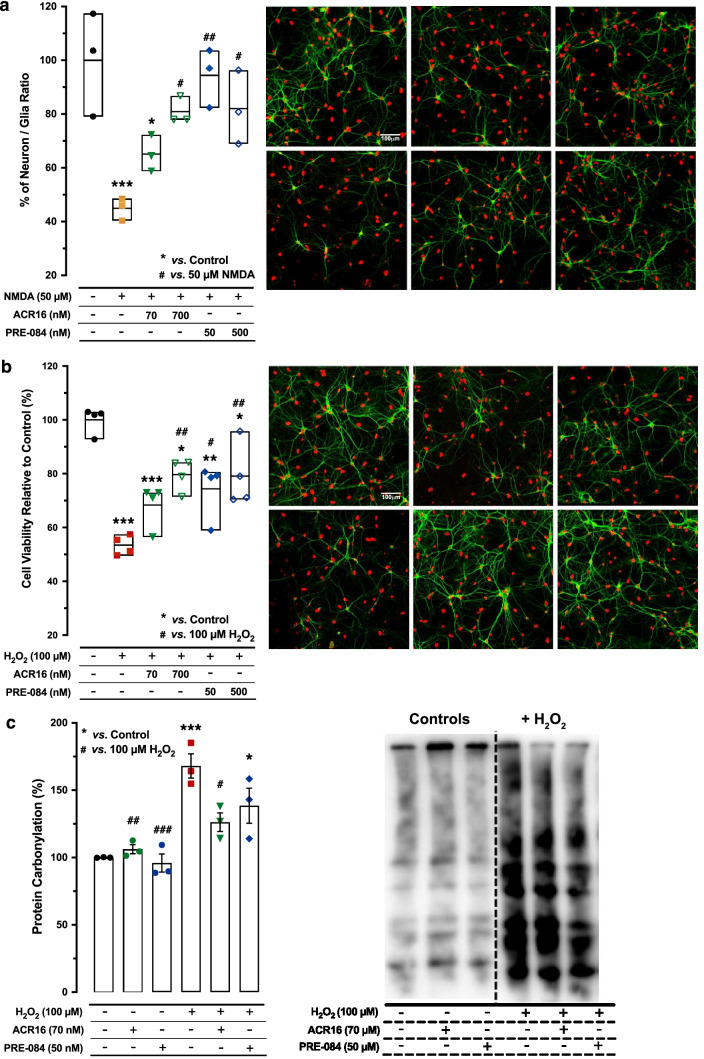
ACR16 and PRE-084 protect against cell death from oxidative stress and NMDA-related toxicity in primary hippocampal cultures. **a** Left: quantitative summary of neuronal survival in NMDA-induced toxicity model and following 24 h of exposure (*n* = 3 for each), as indicated. Data are expressed as a percentage over control conditions [expressed as mean (Min–Max)]. Analysis by 1-way ANOVA (*F* (5,12) = 9.485; ***p < 0.001) and Sidak’s multiple comparisons test. Right: confocal fluorescence microscopy images from rat hippocampal primary cell cultures in which neurons were identified and labeled by MAP-2 immunocytochemistry (green) and cell nuclei were stained with DAPI (pseudocolored in red). Scale bar = 100 μm. **b** Left: quantitative summary that expresses cell viability as the percentage of cell densities relative to those in control conditions following 24 h of exposure [mean (Min–Max); *n* = 4 for each), as indicated. Data were analyzed using 1w-ANOVA (*F*(5,18) = 14.75; ***p < 0.001) and Sidak’s multiple comparisons test. Right: confocal fluorescence microscopy images from rat hippocampal primary cell cultures following 24 h of exposure to different treatments in the H_2_O_2_-induced toxicity model, as indicated. Neurons were identified by MAP-2 immunocytochemistry (green), while cell nuclei were stained using DAPI (red). Scale bar = 100 μm. Significant differences compared with respect to untreated controls are displayed as *, **, and *** for *p* < 0.05, *p* < 0.01, and *p* < 0.001, respectively. Similarly, comparisons again toxicity treatments alone, NMDA (**a**) or H_2_O_2_ (**b**), are shown as #, ##, and ### indicating significant differences of *p* < 0.05, *p* < 0.01, and *p* < 0.001, respectively. Note that the difference between * and # was also indicated directly in the panel. **c** Left: quantitative summary of carbonylated status of proteins in 12 DIV rat hippocampal primary cell cultures following exposure of the indicated treatments for 24 h (mean ± SEM; *n* = 3 for each). Right: representative immunoblot using the Oxyblot kit for detection of carbonylated proteins. Analysis by 2-way ANOVA (*F* (2,12) = 4.889; *p < 0.05) followed by Tukey’s multiple comparisons test was used here for data analysis. Difference between * and # was also indicated directly in this panel

For NMDAR-mediated toxicity studies, 50 µM NMDA was added to 12 DIV primary hippocampal cultures for 24 h, in the presence or absence of σ1R ligand. Neurons are the only cells susceptible to damage from exposure to high concentrations of NMDA [26]. Therefore, cell culture survival was quantified and represented as the ratio between surviving neurons and astrocytes, the neuron/astrocyte ratio, following literature in the field [27]. Neurons and astrocytes were distinguished by immunocytochemistry using neuron-specific MAP2 immunoreactivity, while the total number of cells was identified using cell nuclei stained with DAPI (Suppl. Fig. 1). Each cell type was counted using the imaging software FIJI (National Institutes of Health, NIH) and nuclei that were close to each other forming clusters were separated automatically into individual elements using the FIJI watershed tool [28]. The criteria used here were to define healthy living neurons as those with DAPI-labeled nuclei between 50 and 500 μm^2^ together with neuron-specific MAP2B immunoreactivity, as well as to exclude nuclei with a surface area of fewer than 50 µm^2^ because they were considered cellular debris or neurons with pyknotic nuclei (see Fig. 1 and Suppl. Fig. 2). Unlike NMDA, H_2_O_2_ toxicity produces both neuronal and glial death [24]. Consequently, in this toxicity model, survival was expressed as a percentage of total cell density (number of surviving neurons and astrocytes) to control conditions. Again, after H_2_O_2_ treatment, we quantified healthy living cells as those with DAPI-labeled nuclei with an absolute size between 50 and 500 μm^2^, together with neuron-specific MAP2B immunoreactivity (Fig. 1 and Suppl. Fig. 2). Using this MAP2B neuronal specific marker, we have specifically determined the neuronal survival after H_2_O_2_ treatment and both H_2_O_2_ + ACR16 and H_2_O_2_ + PRE-084 treatments by quantifying the number of MAP2B positive cells per mm^2^ (Suppl. Fig. 4). Oxidative stress was produced by the addition of 100 µM H_2_O_2_ to confluent 12 DIV primary hippocampal cultures for 24 h, in the presence or absence of σ1R ligand. At the end of each experiment, 24 h after insult, primary cell cultures were fixed using 4% paraformaldehyde (PFA) and processed using immunocytochemical techniques. All quantifications were performed on nine arbitrary square fields (600 µm^2^ each) from three independent experiments for each experimental condition and for each method (NMDA, H_2_O_2_) of neuronal toxicity.

### Quantification of Synapses and Dendritic Spines

Both GFP-actin-transfected and non-transfected primary hippocampal cell cultures were used to evaluate the possible synaptogenic and spinogenic effects of σ1R ligands. In the figure legends, in the results section, the cell culture type used for each case is indicated. In all cases, primary hippocampal cultures were treated with one of each σ1R ligand or the vehicle for 48 h. Synapses were identified in cultured neurons at 12 *DIV* using the immunoreactivity of two presynaptic markers: VGLUT1 and synapsin1. These are proteins related to synaptic function used to provide enhanced accuracy and confidence in the quantification of synapses [29, 30]. Using confocal microscopy images analyzed with FIJI software (NIH), we counted manually positive puncta resulting from the merge of VGLUT1 and synapsin1 as individual synapses. Synapse density was calculated as the number of synapses contained in 100 μm length of proximal dendrites, which extend from the soma of clearly identified neurons [21]. A total of 5 independent experiments were performed for each treatment condition, analyzing between 5 and 7 neurons for each treatment and assay. Then, obtained data were normalized to the control conditions, and the variation in the number of synapses was calculated as the average percent change of each treatment condition.

On the other hand, the quantification of individual dendritic spines was performed in a similar manner but at 21 DIV. Here, we used a *bona fide* dendritic spine marker, spinophilin [31], together with VGLUT1 immunoreactivity. The double immunocytochemical detection of VGLUT1, to label specifically synapses established by axon terminals on dendritic spines, and spinophilin, to evaluated spine number, allows the identification of individual synapses at dendritic spines. Here, individual synapse was counted and quantified as positive dots where VGLUT1 and spinophilin immunofluorescence overlapped in clearly identified dendrites. To reduce variability between different primary cell cultures and among treatments, these dots were only counted in proximal dendrites extending up to 100 µm from a clearly identified neuronal soma [21]. Once again, four to six neurons per treatment condition and experiment were randomly analyzed for a total of 3 independent trials. Data were expressed as the density of spinophilin-VGLUT1 puncta per micrometer. Spine density and the number of synapses per spine were evaluated in primary cell cultures at 21 DIV that were previously transfected with EGFP. For the spine density, primary cell cultures were fixed at the end of 48 h of treatment to perform immunocytochemistry and obtain the images using a confocal microscopy. The number of spines was manually counted from confocal images in proximal dendrites using FIJI (NIH) to finally express the spine density as the number of spines per micrometer of dendrite. To this end, between 4 and 7 neurons per experiment and treatment were analyzed from a total of 3 individual experiments. In addition, we carried out a morphometric analysis of spines in control and after σ1R ligand treatment conditions. Spines were first categorized as spines with a neck (thin and mushroom spines) or without a clear neck (stubby spines). Each spine was selected, duplicated, rescaled, and thresholded to identify individual spines and to define the perimeter of the head, splitting it from the neck or the dendritic shaft, to obtain area measurements. Whereas thin spines present a head enlargement less than two-fold the thickness of the neck and with a filiform shape, mushroom spines are distinguished by a head enlargement above two-fold the neck thickness and are mushroom shaped. Regarding the number of synapses per spine, primary hippocampal cultures transfected with EGFP were fixed at 21 DIV and immunolabeled with anti-synapsin1 and anti-EGFP. Images obtained by confocal microscopy were analyzed with FIJI (NIH) for quantification. Discrete fluorescent signal from synapsin1 puncta per spine was manually counted for at least twenty-two dendritic tree fragments from five to seven cells for each of the three independent experiments and treatment conditions.

### Immunocytochemistry and Image Analysis

At the times indicated for each case, the same immunocytochemistry protocol was followed to detect multiple neuronal markers in primary neuronal cultures at 12 DIV or 21 DIV, as previously described [20]. Briefly, at experimental endpoint, neuronal cultures were rinsed with PBS prior to fixation with 4% paraformaldehyde in PBS for 30 min. Then, coverslips were washed three times in PBS and incubated 30 min in blocking solution (2% normal goat serum, 2% BSA, and 0.1% Triton-X 100). Subsequently, coverslips were incubated overnight at 4 °C with primary antibody diluted to the appropriate concentration in blocking solution. Coverslips were washed three times in PBS before the addition of fluorescently conjugated secondary antibody for 30 min at RT in PBS. Samples were then washed five times with PBS and mounted onto slides using Mowiol mounting media containing 1,4-diazobicyclo-[2.2.2]-octane (DABCO) and DAPI. Fluorescence images were obtained using confocal microscope and analyzed using FIJI software. The concentrations at which the primary antibodies were used as follows: guinea pig anti-VGLUT1 (1:1000), rabbit anti-synapsin1 (1:100), rabbit anti-spinophilin (1:800), rabbit anti-MAP2 (1:50), and rabbit anti-GFP (1:5000).

### Animals, Genotyping, and Treatment

Experiments were performed using the transgenic (Tg) mouse strain APP/PS1 (B6C3-Tg(APPswe,PSEN1dE9)85Dbo/Mmjax, Jackson Laboratory, Bar Harbor, ME). This Tg animal model express a chimeric mouse/human amyloid precursor protein (Mo/HuAPP695swe) with the Swedish mutation in APP together with a mutated form of human presenilin 1 (PS1-dE9), both are directed to CNS neurons and mimic a development of early-onset AD [32]. Tg and their non-transgenic (nTg) littermates were housed in groups of 4–5 per cage with chow and water ad libitum on a 12 h light/dark cycle in a controlled environment (21–22 °C). Tg mice were maintained on a C57BL/6 J background using hemizygous Tg males and nTg C57BL/6 females. The genotype of each individual was determined by PCR of genomic DNA isolated from either tail or ear biopsies using the primers specified by Jackson Laboratory. Twelve-month-old Tg and nTg female hemizygous mice were randomly assigned to experimental groups prior to initiating experimental treatments, either to the ACR16 (1.5 mg/kg, *i.p.*) or vehicle (0.9% sterile physiological saline, *i.p.*, Ern, Barcelona, Spain) group. nTg wild-type (WT) littermates were used to demonstrate cognitive deficits in Tg mice. All experimental protocols were approved by the Ethical Committee of the University of La Laguna, based on the European Communities Council Directive 2010/63/EU.

### Morris Water Maze Paradigm

The Morris water maze (MWM), a hippocampal-dependent spatial learning and memory paradigm, was used to evaluate σ1R ligands in Tg mice and their nTg littermates. Mice were treated with σ1R ligand, or vehicle, and temporarily returned to their home cage for 15 min prior to starting the MWM behavioral testing each day. Treatment with σ1R ligand, or vehicle, was administered one time per day during the five consecutive days of the MWM paradigm. The room was illuminated using indirect light to avoid any possible reflection in the pool that could negatively affect the automated video tracking system. Briefly, the behavioral paradigm consisted of four consecutive training days followed by a probe test on the fifth day. During the training phase, each animal was provided four discreet trials (using an inter-trial interval of 15 min and a maximum allotted time of 60 s) to swim and find a submerged platform within a circular pool filled with opaque water. During each training day, animals were randomly placed at one of the four cardinal points (N, W, E, or S) of the circular pool, which was randomly alternated for each of the four trials for each animal. Two types of spatial cues were used: color cues and shape cues. The color cues consisted of 3 different colored squares placed on each wall that surrounded the pool. One wall was absent to allow access to the pool. The yellow square was located on the north wall, pink square was placed in the east wall, and blue square was placed in the south wood wall that hid the computer and experimenter. Shape cues were provided as 4 different geometric shapes made from black foam. These shapes were positioned at each cardinal point of the pool. A triangle was placed in the North, a square was placed in the South, a circle in the East and finally, a 5-pointed star in the West. All cues remained in the same positions during the 5 consecutive days of testing. The submerged platform was covered with a white piece of nylon to facilitate the grip of mice to the platform when it was reached. On the fifth day, the submerged platform was removed, and mice were given 150 s to freely swim within the pool to evaluate retention of spatial memory, in which each animal was placed in the pool in the North position. The Any-Maze video tracking system (Stoelting Europe, Ireland) was used to video record and document the activity of mice during the entire paradigm. The Any-Maze software was used to quantify the total distance traveled for each trial as well as the escape latency to reach the submerged platform during the first four training days. For the probe test, all behavioral parameters were extracted from the video recordings and were quantified using the Any-Maze software.

### qPCR Gene Expression Analyses

Following treatment with both ligands, gene expression was evaluated in 12 DIV primary hippocampal cultures and in hippocampus of mice after completion of the behavioral paradigm. Total RNA was isolated using RNA Spin Plus Kit (Real Laboratory, S.L, Valencia, Spain). From mice, total RNA was isolated from hippocampus of each animal. Mice were sacrificed by cervical dislocation 3 h after performing the MWM probe test and the brains were immediately removed. Both hippocampi were dissected out and snap-frozen in liquid nitrogen. Hippocampal tissue was mechanically homogenized (Ultraturrax T25 homogenizer, IKA, Staufen, Germany) and total hippocampal RNA was isolated using the aforementioned kit. In both cases, the quantity and purity of RNA was measured using a spectrophotometer (NanoDrop 2000, Thermo Fisher Scientific). Total RNA (325 ng/sample for primary cell cultures or 1000 ng/sample for hippocampal tissue) was transcribed using iScript cDNA Synthesis kit (Bio-Rad, Hercules, CA). Real-time PCR (qPCR) was performed for the immediate-early genes Arc, c-fos, and Egr1, in addition to synapsin1 (Syn1), spinophilin (Ppp1r9b), σ1R (Sigmar1), and the glutamate receptor ionotropic NMDA type subunit 1 (Grin1). qPCR experiments were performed using iTaq Universal SYBR Green Supermix (Bio-Rad) in a C1000 thermal cycler (Bio-Rad) connected to a CFX96 optical detector module (Bio-Rad). For each gene, each sample was measured in triplicate within the same 96-well plate. mRNA expression was normalized by a geometrical averaging of three internal control genes (glyceraldehyde 3-phosphate dehydrogenase (Gapdh), ribosomal protein L13a (Rpl13a), and cyclophilin A (CycA) following the procedure described in Vandesompele et al. [33]. The stability of internal control genes was measured and verified by their M-values. All of the primers used with primary hippocampal cultures (Table 1a) were provided by DNA technologies (Coralville, IA, USA) and all of the primers used with mouse hippocampi (Table 1b) were purchased from Sigma-Aldrich (St. Louis, MO, USA).

**Table 1:** Sequence of all primers used in qPCR. Primers employed to perform real-time PCR in primary hippocampal cell cultures (a) and APP/PS1 mouse hippocampi (b). Gene symbol, Genebank accession numbers, and their sense or antisense oligonucleotide sequences are shown

**Genes**	**Accession no**	**Forward (5′-3′)**	**Reverse (5′-3′)**
**a)** Primers for primary rat hippocampal cultures			
Rpl13a	NM_173340	GGATCCCTCCACCCTATGACA	CTGGTACTTCCACCCGACCTC
CycA	NM_017101	TATCTGCACTGCCAAGACTGAGTG	CTTCTTGCTGGTCTTGCCATTCC
Gapdh	NM_017008	ATGGGAAGCTGGTCATCAAC	GTGGTTCACACCCATCACAA
Arc	NM_019361	GCCCCCAGCAGTGATTCAT	CACCTGGCTCTGAAGACTCC
c-fos	NM_022197	CCAAGCGGAGACAGATCAACT	AGTCAAGTCCAGGGAGGTCA
Syn1	X04655.1	AGGCTACCCGTCAGGCATCTATCTC	TCACCTCATCCTGGCTAAGG
Ppp1r9b	NM_053474	AGACTGTGACTGAGGGTGGT	GGCCAATCATGAACCGCAC
Sigmar1	NM_030996	GCTGGATGGGCGCCATGTGT	GCCCAGTATCGTCCCGAATGGC
Grin1	NM_001270602	CTGACAAGAGTATCCACCTGAGT	GTCCGCGCTTGTTGTCATAG
**b)** Primers for APP/PS1 mouse hippocampus			
Rpl13a	NM_009438	GGATCCCTCCACCCTATGACA	CTGGTACTTCCACCCGACCTC
CycA	NM_008907	TATCTGCACTGCCAAGACTGAGTG	CTTCTTGCTGGTCTTGCCATTCC
Gapdh	NM_001289726	ATGGGAAGCTGGTCATCAAC	GTGGTTCACACCCATCACAA
Arc	NM_018790	GGCAGCGGCTGGAGCCTACAGAG	GCTCTTGGGCTGAGCTGGGGTGCT
c-fos	NM_010234	GTTTCAACGCCGACTACGAG	TGTCACCGTGGGGATAAAGT
Egr1	NM_007913	ACCACAGAGTCCTTTTCTGACAT	AGCGGCCAGTATAGGTGATG
Syn1	NM_013680	TGAGGACATCAGTGTCGGGTAA	GGCAATCTGCTCAAGCATAGC
Ppp1r9b	NM_172261	ACCGCACCGATCCAAGTATT	GCTCATATTCCGCAGAGGCT
Sigmar1	NM_011014	GCTCGACAGTATGCGGGGCT	CAGACAGCGAGGCGTGCAGA
Grin1	NM_008169	TCATCCTGCTGGTCAGCGATGAC	AGAGCCGTCACATTCTTGGTTCCTG

### Western Blot Immunodetection and Oxidation Status of Proteins

Following treatment, primary hippocampal cultures were lysed as we previously described [20]. Lysates were heated, briefly sonicated, and clarified by centrifugation at 10,000 × *g* at 4 °C. Snap-frozen hippocampal samples from mice were weighed and homogenized in urea buffer 7 M (1 mL/10 mg brain tissue) as described for primary hippocampal cultures above. The BCA method was used to determine total protein content of each lysate and equal amounts of protein were loaded and separated by SDS-PAGE. Proteins were transferred to PVDF membranes for Western blotting. PVDF membranes were blocked, incubated overnight with primary antibody, and thoroughly washed prior to incubation with HRP-conjugated secondary antibody. The visualization of immunodetected proteins was made using Clarity ECL Substrate (Bio-Rad) and a ImageQuant LAS 500 CCD Camera (General Electric, Boston, MA, USA). Quantification of immunodetected protein was performed on digital images acquired from the imaging software in FIJI (NIH). The following primary antibodies were used: anti-actin (1:5000), anti ERK 1/2 antibody (1:1000), anti-phospho ERK 1/2 (1:1000), anti-Pan-Akt (1:1000), anti-phospho-Akt (1:1000), anti-synapsin1 (1:1000), and anti-spinophilin (1:1000).

Oxidation status of proteins was determined in primary hippocampal cultures at 12 DIV, with or without σ1R ligand, in the presence or absence of 100 µM H_2_O_2_ for 24 h (*n* = 3 for each experimental condition). The Oxyblot Protein Oxidation Detection Kit (Millipore) was employed, providing all the required reagents for the immunodetection of carbonylated proteins. Assays were performed incubating primary hippocampal cell cultures to different experimental treatments for 24 h: 70 nM ACR16, 50 nM PRE-084, 100 μM H_2_O_2_, and combined treatments of 70 nM ACR16 or 50 nM PRE-084 with 100 μM H_2_O_2_. Fresh medium without treatment compounds was added to cultured neurons used as controls. After 24 h of treatment, culture medium was removed, and cells were rinsed two times with ice-cold PBS. Subsequently, cell cultures were lysed using cell lysis buffer (Cell Signaling Technology #9803, USA), and cell lysates were transferred to Eppendorf tubes and incubated for 15 min on ice. Lysates were subsequently centrifuged at 14,000 rpm at 4 °C for 20 min, prior to processing them using the manufacture’s protocol. Finally, PVDF membranes were incubated with Clarity ECL Substrate and the chemiluminescence was imaged using a ImageQuant LAS 500 CCD Camera. FIJI software was employed for densitometry calculations. The data were normalized to basal (control) conditions, and the amounts of carbonylated proteins were expressed as the percent change from basal levels.

### Statistics

All data were graphed and analyzed using GraphPad Prism software package. Data were expressed as mean ± SEM, mean ± min to the max or median ± min to the max as indicated in the corresponding figure legend. Data from in vitro models were analyzed using a 1-way ANOVA followed by a post hoc Sidak’s multiple comparisons test, except for the protein carbonylation and protein expression temporal dynamics. For these cases, data were analyzed by 2-way ANOVA using a post hoc Tukey’s multiple comparisons test for the two first cases and a Sidak’s post hoc test for temporal dynamics. In addition, gene expression studies in cell cultures were analyzed using a Student’s *t* test. For animals, data were analyzed by Student’s *t*-test or 2-way ANOVA with a Sidak’s post hoc test, as indicated in the figure captions.

## Results

### ACR16 and PRE-084 Prevent Neuronal Toxicity

Neuronal cell injury was produced by excitotoxicity in primary hippocampal cultures at 12 DIV by exposure to 50 µM NMDA for 24 h. Cell cultures were subsequently fixed and fluorescently immunolabeled for MAP-2 expression, which is specific to neurons. Neuronal survival was calculated 24 h later as the ratio between surviving neurons and astrocytes [mean ± SEM (Min–Max)], which was reduced by more than 50% by 50 µM NMDA alone [44.96% ± 2.4 (40.4–48.47)] when normalized and compared to control conditions [100.0% ± 11.2 (79.07–117.3)] (Fig. 1a). The addition of the σ1R ligand ACR16 at both a low (70 nM) and an intermediate (700 nM) concentration attenuated NMDA-mediated excitotoxicity and increased neuronal survival to 65.14% ± 3.9 (58.76–72.27) and 80.86% ± 2.9 (77.92–86.71), respectively (Fig. 1a). A similar effect was observed with the σ1R agonist PRE-084, in which 50 µM NMDA produced less than 20% reduction in neurons, respective of the concentration of PRE-084 [94.37% ± 6.3 (82.38–103.7) and 82.02% ± 7.9 (68.94–96.3), for 50 nM and 500 nM] (Fig. 1a). We have also discarded any effect due to ACR16 and PRE-084 by confirming that in the absence of NMDA injury neither of the two σ1R ligands affected the neuron/astrocyte ratio at high doses (Suppl. Fig. 3a). Finally, we have also confirmed that NMDA, NMDA + ACR16, and NMDA + PRE-084 treatments yield no effect on astrocyte number (data not shown).

The abilities of each of these σ1R ligands were also evaluated with regard to cellular oxidative stress produced by the addition of H_2_O_2_ to culture medium, a common cellular toxicity model. Unlike NMDA, oxidative stress produced by H_2_O_2_ is not only specific to neurons but affects both neurons and glia in mixed primary cell cultures. For this reason, in these experiments, cellular density [mean ± SEM (Min–Max)] was used as a way to evaluate neuronal survival. Here, primary hippocampal cultures at 12 *DIV* were treated for 24 h with 100 µM H_2_O_2_, either alone or in combination with low or high concentrations of each σ1R ligand. Again, 24 h later, cell cultures were fixed and fluorescently immunolabeled for MAP-2 expression and the total cell density was calculated. Total cell density was reduced up to 47% by 100 µM H_2_O_2_ [53.42% ± 1.8 (49.66–57.47)] when normalized and compared to control conditions (100.0% ± 2.4 (92.76–103.0)) (Fig. 1b). In addition to providing protection from NMDA-mediated excitotoxicity, both σ1R ligands prevented the decrease in cell density by 100 µM H_2_O_2_. A clear concentration–response was observed with ACR16 [68.29% ± 4.0 (56.45–72.88) *vs.* 79.59% ± 3.0 (71.41–84.18), for the low (*n* = 70 nM) and high (700 nM) concentrations, respectively] while both concentrations of PRE-084 prevented cellular death to a similar extent [74.38% ± 5.2 (58.96–80.65) and 79.09% ± 5.9 (70.40–95.76)] (Fig. 1b). Again, as a control, we then evaluated whether ACR16 and PRE-084 have any effect on cellular density in primary hippocampal cultures, without 100 µM H_2_O_2_ injury_._ Our data indicate that neither ACR16 nor PRE-084 treatments at high doses affected total cell density (Suppl. Fig. 3b). To address the specific effect on neuronal survival in the H_2_O_2_ experiments, we have also quantified the number of MAP-2 positive neurons after H_2_O_2_ treatment and in combination with ACR16 and PRE-084. Surviving neurons [mean ± SEM (Min–Max)], were reduced to a 18.24% after treatment with 100 µM H_2_O_2_ alone [17.35 ± 4.96 neurons/mm^2^ (6.09–28.44)] when compared to control conditions [95.08 ± 11.42 neurons/mm^2^ (72.32–126.21)] (Suppl. Fig. 3c). Here, the addition of ACR16, at both a low (70 nM) and an intermediate (700 nM) concentration increased neuronal survival to a 54.39% and 63.02% [51.71 ± 7.71 neurons/mm^2^ (39.12–65.73) and 59.92 ± 8.81 neurons/mm^2^ (39.45–82.12)], respectively (Suppl. Fig. 3c). In turn, PRE-084 at both a low (50 nM) and an intermediate (500 nM) concentration increased neuronal survival to a 47.37% and 65.59% [45.04 ± 10.63 neurons/mm^2^ (32.11–66.11) and 62.36 ± 4.93 neurons/mm^2^ (35.24–80.47)], respectively (Suppl. Fig. 3c).

To further explore the protective effect by both σ1R ligands in H_2_O_2_-dependent oxidative stress, we evaluated of the oxidation status of cellular proteins by protein carbonylation after a 24 h exposure to 100 µM H_2_O_2_ (mean ± SEM). Protein carbonylation is an irreversible oxidative modification induced by oxygen free radicals and other reactive species [34]. Evidence indicates that elevated protein carbonylation levels are a consequence of acute, or prolonged, oxidative stress link to the etiology and progress of age-related diseases, as AD [35]. Therefore, these protein forms are commonly used as an indicator of protein oxidative stress and aging [36]. Since both compounds, at their respective low concentrations, significantly prevented the reduction in cell density by 100 µM H_2_O_2_, these low concentrations were evaluated. In primary hippocampal cultures at 12 *DIV*, the extent of carbonylated proteins was determined after a 24 h exposure to 100 µM H_2_O_2_ (Fig. 1c). In the absence of oxidative stress, neither ACR16 nor PRE-084 modified the extent of total carbonylated proteins (Fig. 1c), while they were significantly increased in primary cells treated with 100 µM H_2_O_2_. ACR16 and PRE-084 reduced carbonylated proteins by approximately 42% (126.2% ± 6.9; *n* = 3) and 30% (138.5% ± 13.0; *n* = 3), respectively (Fig. 1c), and compared to the toxicity control conditions (168.0% ± 9.0; *n* = 3). However, this change is only significant for ACR16. Together, these toxicity studies indicate that PRE-084, but particularly ACR16, reduces the accumulation of ROS during oxidative stress, providing neuroprotection.

### ACR16 and PRE-084 Modulate Synapse Number

**Fig. 2:**
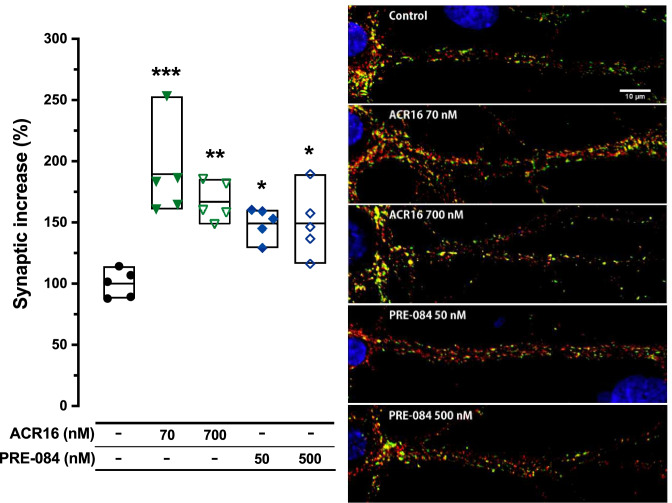
ACR16 and PRE-084 increase the number of synapses in 12 DIV rat hippocampal primary cell cultures*.* Confocal fluorescence microscopy of immunolabeled hippocampal primary cell cultures following 48 h exposure to different concentrations of σ1R agonists, as indicated; VGLUT1 (red) and synapsin1 (green), scale bar = 10 μm. The discrete areas with double-immunoreactivity were identified as synapses and quantified. The adjacent graph displays the results of the count for each experimental condition and each individual experiment [mean (Min–Max); *n* = 5]. Results are expressed as the mean ± min to the max, where each dot represents a single experimental value. Statistical analysis was performed using 1-way ANOVA (*F* (4,20) = 10.13; ***p < 0.001) and Tukey’s multiple comparisons post-test. Significant differences compared with control controls are expressed as *(*p* < 0.05), **(*p* < 0.01), and ***(*p* < 0.001), respectively

To examine whether σ1R agonists play a role in synaptogenesis, changes in the number of synapses were monitored in primary hippocampal cultures at 12 DIV following exposure to low, or high, concentrations of ACR16 or PRE-084. After 48 h, cell cultures were fixed and fluorescently immunolabeled for VGLUT1 and synapsin1 to detect and facilitate the quantification of synapses (Fig. 2). The detection of double immunoreactivity from both presynaptic VGLUT1 and synapsin1 markers serves to unambiguously identify a well-established synapse. Fiji software was used for synaptic density quantification [mean ± SEM (Min–Max)] (see the “Methods” section). A highly significant increase was observed in the number of synapses of neurons treated with a low or high concentration of ACR16, a 90% [189.5% ± 16.7 (160.6–253.1); 24 dendrites from *n* = 5 distinct cultures] or 67% [166.9% ± 7.1 (148.7–185.5); 23 dendrites from *n* = 5 distinct cultures] increase when compared to control conditions [100.0% ± 5.1 (87.9–114.3); 26 dendrites from *n* = 5 distinct cultures]. A similar effect was also observed with PRE-084, which yielded slightly lower increases in synapse number at both concentrations compared to control conditions than the levels observed with ACR16 [149.4% ± 5.8 (129.1–160.4); 32 dendrites from *n* = 5 distinct cultures, and 149.2% ± 12.1 (116.2–189.4); 22 dendrites from *n* = 5 distinct cultures], for the low and high concentrations, respectively (Fig. 2). These results suggest a synaptogenic role for both σ1R ligands evaluated, in which ACR16 was found to be more synaptogenic than PRE-084.

### ACR16 and PRE-084 Induce Spinogenesis

**Fig. 3:**
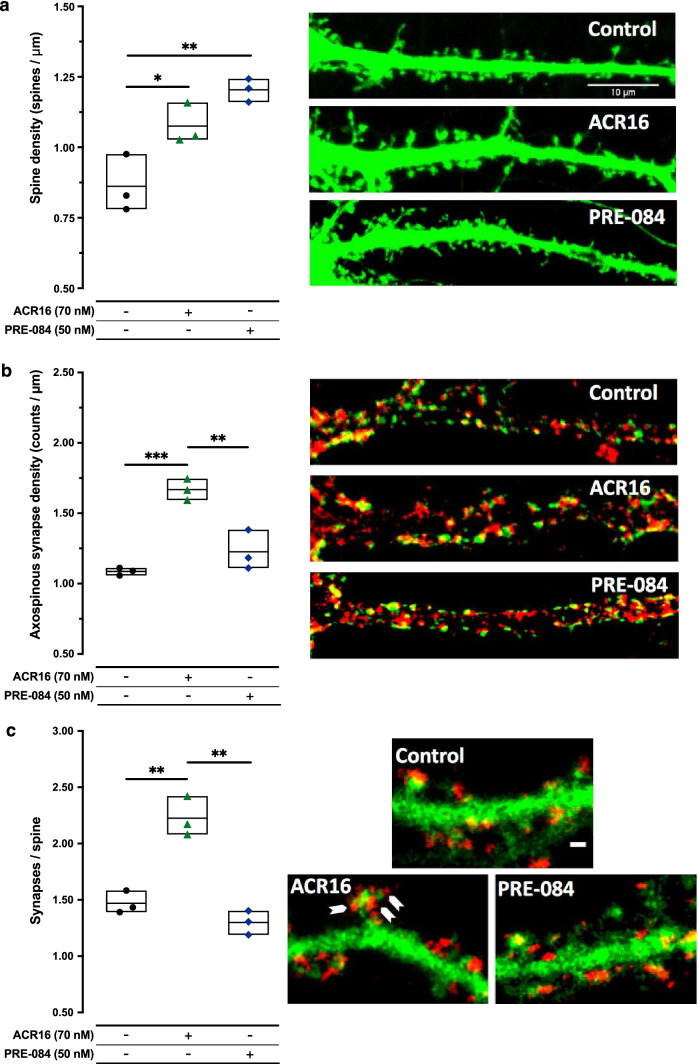
ACR16 and PRE-084 treatments stimulate new dendritic spine formation but only ACR16 generates more axospinous synapses and increases the number of synapses per spine. **a** Left: quantitative summary of the number of spines per unit length (μm) for each experimental condition (*n* = 3). A significant increase was found compared to control conditions for ACR16 and PRE-084, *(*p* < 0.05) and **(*p* < 0.01), respectively. Right: confocal fluorescence microscopy images of proximal dendrites (~ 50 μm in length) taken from 21 DIV rat hippocampal primary cell cultures transfected with EGFP in different experimental conditions, as indicated, following 48 h of treatment in which spines are observed (scale bar = 10 µm). **b** Left: quantitative summary of the number of double-immunoreactive areas (yellow) per unit length (µm) for each treatment condition. Significant differences are indicated as ***(*p* < 0.001) when ACR16-treated are compared to control cultures and **(*p* < 0.01) when ACR16-treated are compared to PRE-084-treated cultures. Right: confocal fluorescence microscopy images of 21 DIV hippocampal primary cell cultures with immunoreactivity for VGLUT1 (red) and spinophilin (green) after exposure to different experimental conditions for 48 h, as indicated (scale bar = 10 µm). **c** Left: graph expressing the average density of immunoreactive dots per spine and experimental condition. Significant differences are indicated as **(*p* < 0.01) when ACR16-treated are compared to control cultures and **(*p* < 0.01) when ACR16-treated are compared to PRE-084-treated cultures. Right: confocal fluorescence microscopy of synapsin1 (red) and EGFP-transfected hippocampal primary cell cultures at 21 *DIV* after exposure to σ1R agonists for 48 h. Images exhibit a portion of proximal dendrites of neurons, where synapsin1 immunoreactive dots (synapses) are observed on EGFP-expressing spines. Arrows indicate multiple synapses on one dendritic spine. Data analysis for **a** (*F* (2,6) = 15.56; **p < 0.01), **b** (*F* (2,6) = 31.51; ***p < 0.001), and **c** (*F* (2,6) = 41.61; ***p < 0.001) included 1-way ANOVA followed by Tukey’s multiple comparisons test

Prompted by the strong synaptogenic effect observed by both σ1R ligands, we also evaluated the possible involvement of ACR16 and PRE-084 in modulating dendritic spine density. Primary cultures of neurons expressing EGFP were used to facilitate the visualization and identification of spines along dendrites. Based on the strong synaptogenic effect of low concentrations of σ1R ligand, only the lower concentrations of ACR16 (70 nM) and PRE-084 (50 nM) were evaluated. Primary hippocampal cultures at 19–21 *DIV* were treated with or without σ1R ligand for 48 h, subsequently fixed and imaged using confocal microscopy to quantify the number of spines per micrometer [mean ± SEM (Min–Max)]. Both ligands produced significant increases in the number of spines per micrometer of dendrite (Fig. 3a). Primary cell cultures in control conditions presented an average of 0.862 ± 0.06 (0.781–0.976) spines per micrometer while neurons treated with ACR16 at 70 nM exhibited 1.076 ± 0.04 (1.027–1.159) spines per micrometer, meaning an approximate 25% increase compared to control conditions. Interestingly, the low concentration of PRE-084 produced a greater increase in spine density than ACR16 [1.204 ± 0.02 (1.161–1.243) spines per micrometer, corresponding to an increase of approximately 40% compared to control conditions; 24 dendrites from each treatment, *n* = 6 distinct cultures] (Fig. 3a). We then carried out a morphological analysis to determine how ACR16 and PRE-084 modulate spine morphology (Table 2). Spine type distribution were different between control and treated (ACR16/PRE-084) cultures. Stubby spine number were higher in ACR16- and PRE-084-treated neurons when compared to control whereas control cultures showed larger number of mushroom spines with respect to treated cultures (Table 2). Interestingly, we observed a higher percentage of stubby spines in PRE-084 [39.70 ± 0.89 (20 proximal dendrites from *n* = 3 distinct cultures)] compared to ACR16 [23.59 ± 0.57 (20 proximal dendrites from *n* = 3 distinct cultures)] and control [17.01 ± 0.89 (20 proximal dendrites from *n* = 3 distinct cultures)]. Regarding thin spines, no differences were detected between any group (Table 2). However, when we analyzed mushroom spines, we have detected a higher percentage of this spine type in ACR16 [58.91 ± 1.06 (20 proximal dendrites from *n* = 3 distinct cultures] compared to PRE-084 [42.60 ± 1.89 (20 proximal dendrites from *n* = 3 distinct cultures] treatments (Table 2).

**Table 2:** Morphometric analysis of spines. Spine morphological analysis in control and after ACR16 (70 nM) and PRE-084 treatments evaluating relative amount (percentage (%), mean ± SEM) of stubby spines (no clear neck) thin and mushroom spines (both presenting a neck). An analysis by Student’s *t*-test was used here for data analysis. Significant statistical differences are as follows: (a) stubby spines, ACR16 compared to control: ***(*p* < 0.001), PRE-084 compared to control: ***(*p* < 0.001), PRE-084 compared to ACR16: ***(*p* < 0.001), (b) mushroom spines, ACR16 compared to control: ***(*p* < 0.001), PRE-084 compared to control: ***(*p* < 0.001), PRE-084 compared to ACR16: ***(*p* < 0.001). No significant differences were observed for thin spines

	**Spine classes**		
	Stubby (mean ± SEM)	Thin (mean ± SEM)	Mushroom (mean ± SEM)
Control	17.01 ± 0.33	15.45 ± 0.52	67.56 ± 0.55
ACR16 (70 nM)	23.59 ± 0.57	17.49 ± 0.94	58.91 ± 1.06
PRE-084 (50 nM)	39.70 ± 0.89	17.72 ± 1.29	42.60 ± 1.17

Due to the clear spinogenic effect, we investigated the correlation between dendritic spines and synapses. Again, using primary hippocampal cultures at 19–21 *DIV*, neurons were treated with or without the low concentrations of σ1R ligand for 48 h, and then fixed, and fluorescently immunolabeled for spinophilin and VGLUT1. To reduce variability among distinct cultures and treatments, synaptic puncta were exclusively analyzed in proximal dendrites, starting from a clearly identified neuronal cell soma. In these proximal dendrites, discrete areas in which VGLUT1 and spinophilin immunofluorescence overlapped were identified, quantified, and represented as a ratio of spinophilin to VGLUT1 signal (Fig. 3b). Spine density was represented as the number of VGLUT1-spinophilin positive dots per 100 µm of dendritic length [mean ± SEM (Min–Max)]. ACR16 at 70 nM produced a highly significant increase [1.668 ± 0.04 (1.593–1.746), 21 images from *n* = 4 distinct cultures] while PRE-084 produced a modest but significant increase [1.226 ± 0.08 (1.111–1.383), 13 images from *n* = 4 distinct cultures] compared to primary hippocampal neurons in control conditions [1.086 ± 0.02 (1.058–1.111), 17 images from *n* = 4 distinct cultures] (Fig. 3b). These results suggest that only ACR16 produces spines involved in the formation of new synapses. We also investigated the relative number of synapses per dendritic spine using neurons expressing EGFP. Primary hippocampal cultures at 19–21 *DIV* were treated with or without the low concentrations of σ1R ligand for 48 h, and subsequently fixed and fluorescently immunolabeled for synapsin1. All synapsin1-reactive puncta on dendritic spines were identified, counted, and quantified [mean ± SEM (Min–Max)] along the primary proximal dendrites of EGFP-expressing neurons (Fig. 3c). In control conditions, the average number of synapses per spine was 1.469 ± 0.06 (1.391–1.583; 24 images from *n* = 3 distinct cultures), while ACR16 at 70 nM produced a significant increase in synapses per spine [2.225 ± 0.10 (2.081–2.421); 24 images from *n* = 3 distinct cultures] (Fig. 3c). Interestingly, the low concentration of PRE-084 did not alter the number of synapses per spine [1.299 ± 0.06 (1.189–1.402); 24 images from *n* = 3 distinct cultures] (Fig. 3c). Taken together, both σ1R ligands promote new spines formation in primary hippocampal neurons, but only ACR16 increases the number of new synapses per spines.

### Induction of PI3K and ERK Signaling Cascades by PRE-084 and ACR16 and Their Effect on Synaptic Protein Expression

**Fig. 4:**
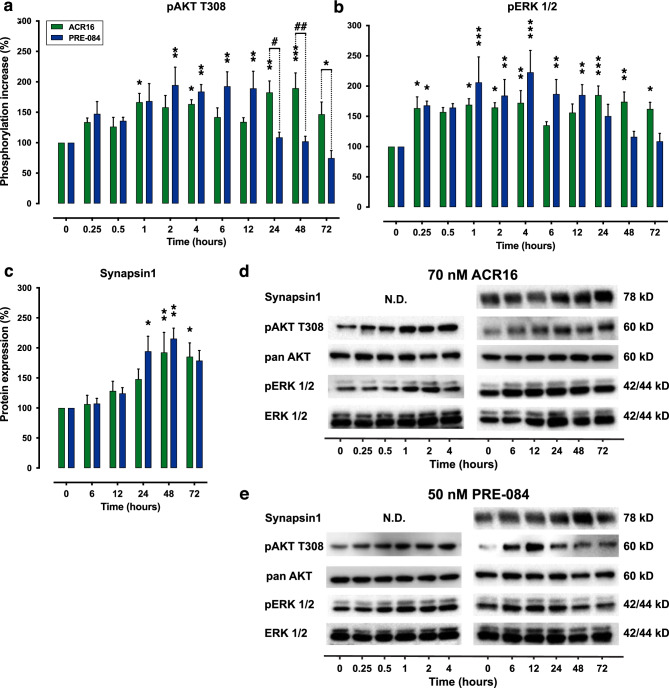
ACR16 and PRE-084 increased synapsin1 protein expression and activated both MAPK and PI3K signaling pathways, in which ACR16 maintained this activation over a prolonged period*.* Rat hippocampal primary cell cultures were treated with 70 nM ACR16 or 50 nM PRE-084 at 12 DIV, and the phosphorylation status of AKT and ERK 1/2 was quantified using Western blot (**a** and **b**), in addition to protein expression of synapsin1 (**c**). An extended time-course, from 15 min to 72 h, was used to study short and longer cellular events. Data were normalized to control conditions and expressed as the percentage change of pAKT (**a**) or pERK 1/2 (**b**) normalized to pan AKT or ERK1/2 protein respectively, as well the percentage change of protein expression of synapsin1 normalized to pan AKT (**c**). 2-way ANOVA followed by Sidak’s multiple comparisons test was used for data analysis, comparing the percent change over time versus untreated control basal levels at time point 0, as well as between treatments. Significant differences compared with untreated controls are displayed as *(*p* < 0.05), **(*p* < 0.01), and ***(*p* < 0.001), respectively. Significant differences between ACR16 and PRE-084 treatments are shown as #(*p* < 0.05) and ##(*p* < 0.01). Representative Western blots are different time points are presented in **d** and **e**

σ1R activation is known to trigger downstream targets of PI3K-Akt and ERK-MAPK signaling cascades [37]. Here, we verified the activation of ERK and AKT signaling pathways using the low concentrations of ACR16 (70 nM) and PRE-084 (50 nM) in primary hippocampal cultures (Fig. 4). The temporal dynamics of AKT and ERK 1/2 were measured by Western blot in primary cell cultures, using an extended time-course ranging between 15 min (0.25 h) and 72 h (Fig. 4d–e). The temporal evaluation of σ1R agonists indicated that both gradually increased the phosphorylation of AKT at position T308, which is a critical phosphorylation site for the AKT activation [38], reaching significant levels over baseline phosphorylation after 1 h of exposure to treatments (Fig. 4a), although these agonists exhibited different temporal dynamics (*p* < 0.001; 2-way ANOVA with Sidak’s post hoc test). While 50 nM PRE-084 produced a significant increase in AKT phosphorylation that was sustained between 2 and 12 h, 70 nM ACR16 exhibited two phosphorylation phases or waves; the first one occurred from 1 to 4 h after exposure to ACR16, while the second wave was much longer that began at 24 h and was maintained until 72 h, the last time point evaluated (Fig. 4a).

We found similar dynamics with phosphorylated ERK1/2 at residues T202/Y204 (*p* < 0.01; 2-way ANOVA with Sidak’s post hoc test). The addition of 70 nM ACR16 produced two peaks of ERK 1/2 phosphorylation. The first appeared 15 min after exposure and lasted up to 4 h. The subsequent wave of phosphorylated ERK 1/2 occurred between 24 and 72 h after ACR16 exposure (Fig. 4b). In contrast, PRE-084 only provided a single phosphorylation phase, where phosphorylated levels of ERK 1/2 significantly increased from the first hour of drug exposure until up to 12 h (Fig. 4b).

Regarding synapsin1 protein expression levels, the effects produced by the addition of 70 nM ACR16 or 50 nM PRE-084 to 12 DIV primary hippocampal cell cultures were also evaluated. Here, both agonists increased synapsin1 protein expression (*p* < 0.001; 2-way ANOVA with Sidak’s post hoc test) to reach the maximal expression at 48 h of exposure (Fig. 4c). Although, it was found that PRE-084 produces an earlier increase than ACR16; at 24 h *versus* 48 h, respectively (from *n* = 4 independent cultures, with 2 replicates per culture). Additionally, no changes in synapsin1 protein expression were observed following sigma1 ligand treatment at earlier time points (15 min to 4 h; data not shown).

### ACR16 and PRE-084 Modify Selective Gene Expression in Primary Cell Cultures

**Fig. 5:**
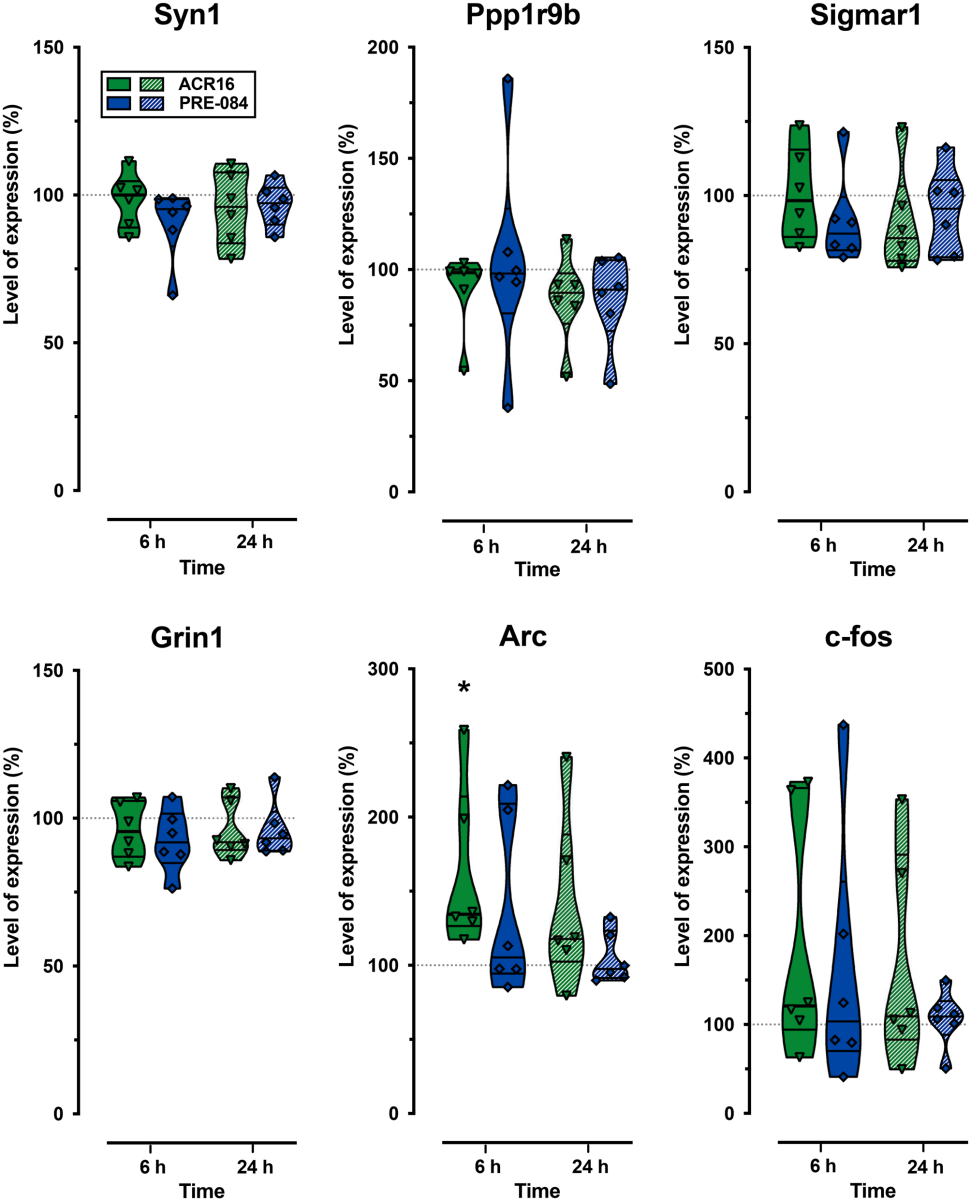
The σ1R agonist ACR16 increased Arc gene expression. The expressions of synapsin1 (Syn1), spinophilin (Ppp1r9b), σ1R (Sigmar1), subunit 1 NMDAR (Grin 1), and the immediate-early genes Arc and c-fos were quantified in rat hippocampal primary cell cultures using qPCR. Individual data were normalized as percent change and represented in the violin plots as median percent change with respect to control conditions ± (Min–Max). Six independent experiments were performed for each experimental condition and for each time point. Statistical analysis was performed using Student’s *t*-test. Comparisons were made with control cultures and between treatments and *(*p* < 0.05) is used to indicate significant differences to control cultures. Note that only ACR16 (70 nM) was found to increase Arc expression at 6 h

Based upon the observations mentioned above, we explored possible changes in the expression of several genes linked to synaptic plasticity, as well as the σ1R coding gene (Sigmar1). Syn1 and Ppp1r9b are the coding genes for synapsin1 and spinophilin, two structural proteins involved in synaptic plasticity. Synapsin1 regulates the trafficking of synaptic vesicles and neurotransmitter release, while spinophilin is implicated in spine morphology and density regulation [39, 40]. Grin1 gene encodes the glutamate ionotropic receptor NMDA type subunit 1, a critical subunit of these essential mediators of synaptic transmission and plasticity [41]. We also analyzed the expression of the immediate-early genes Arc and c-fos, whose upregulation is necessary during learning and memory recall [42]. In addition, Arc has been linked to AD pathophysiology and synapse loss [43]. To this end, primary hippocampal cultures at 12 DIV were treated with low concentrations of ACR16 or PRE-084 for 6, or 24 h, before determining gene expression levels by qPCR. No significant change was observed in the expression of studied genes, except for the immediate early gene Arc, where only ACR16 (70 nM) was found to increase its expression at 6 h (134.4%; 95% *CI*, 104.2 to 220.2; *n* = 6; *p* < 0.05) (Fig. 5). The absence of changes in Arc gene expression by PRE-084 suggests considerable differences in the mechanisms that underlie the effects of both agonists.

### ACR16 Improves Spatial Learning and Memory in APP/PS1 Mice

**Fig. 6:**
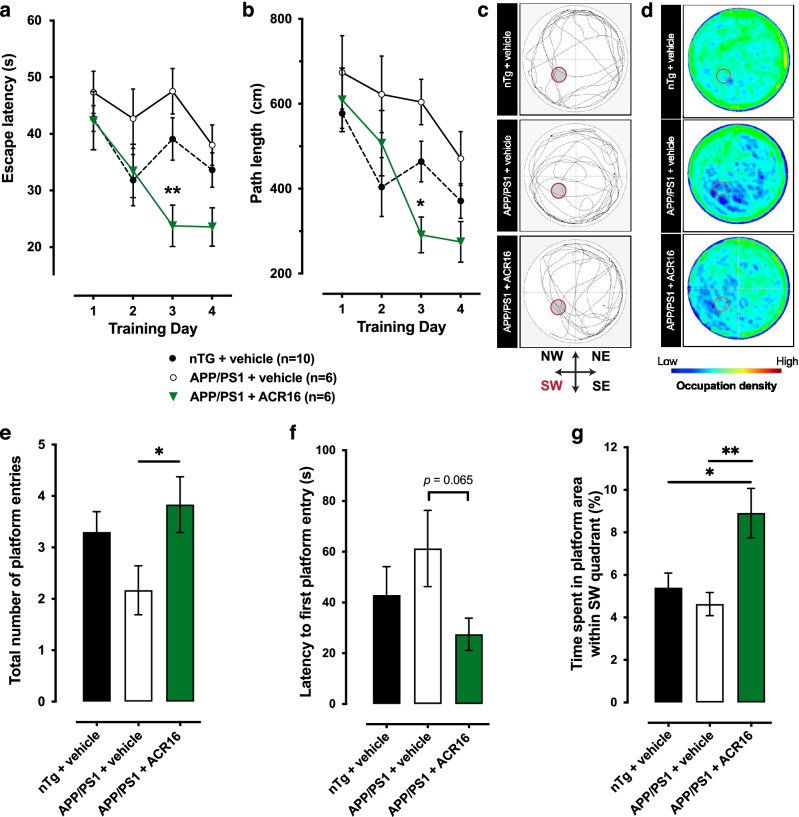
ACR16 reduces spatial memory deficits associated with the APP/PS1 transgenic model of AD. **a** Comparison of the average latency to discover the submerged platform for nTg mice (*n* = 10), saline-treated APP/PS1 mice (*n* = 6), and APP/PS1 mice treated with 1.5 mg/kg ACR16 (*n* = 6). Both nTg mice and APP/PS1 mice treated with ACR16 exhibit significant differences compared to saline treated APP/PS1 mice, in which less time was required to reach the submerged platform. **b** Average distance traveled to reach the platform. nTg mice and APP/PS1 treated with ACR16 used shorter paths to reach the platform than untreated APP/PS1 mice. A Sidak’s multiple comparisons test was performed after 2-way ANOVA in **a** and **b**, where significant differences as shown as *(*p* < 0.05) and **(*p* < 0.01). Probe test observations are provided as individual swimming paths in the MWM from a representative animal for each group (**c**) and as heat map for the activity of all subjects in each group overlayed onto each other in the MWM (**d**). Here, untreated APP/PS1 mice predominantly swam close to the edge of the pool in circular paths (**d**). Together with less presence in the SW quadrant and the inner part of the pool (blue color areas), these data suggest a great difficulty in remembering the former position of the platform (**d**). In contrast, nTg and ACR16 mice treated with APP/PS1 have a high presence in the central part of the pool and show a higher frequency of passage (green areas) through the area previously occupied by the platform (**d** and **e**). **f** In the probe test, ACR16-treated transgenic mice had shorter times to reach the platform for the first time compared to untreated transgenic mice and wild-type mice. **g** Similarly, to the number of entries, the same trend was observed when comparing the total time spent in the platform area between groups, where treated APP/PS1 mice spent more time in this area than the other groups. Data in **e**, **f**, and **g** are expressed as mean ± S.E.M and analyzed by a Student’s *t*-test. Significant differences are represented as *(*p* < 0.05) and **(*p* < 0.01)

Older Tg APP/PS1 mice exhibit cognitive impairment that affects spatial learning and memory. This impairment can be easily observed and quantified during the performance of the acquisition and recall phases in the MWM. Based on the synaptogenic and spinogenic observations in primary neurons, we evaluated, in 12-month-old Tg APP/PS1 mice, the effect of a low dose of ACR16 (1.5 mg/kg/day) in the performance of the MWM behavioral paradigm. During daily training sessions, we observed that untreated Tg mice required more time (*p* < 0.05; 2-way ANOVA with Sidak’s post hoc test) and traveled longer paths (*p* < 0.05) to reach the submerged platform than their nTg littermates (Fig. 6a, b). The same trend was observed in treated Tg mice. Remarkably, 1.5 mg/kg/day ACR16 significantly improved the MWM performance, reducing the escape latency (*p* < 0.05; 2-way ANOVA with Sidak’s post hoc test) and the total distance traveled to reach the hidden platform (*p* < 0.05) compared to untreated Tg mice (Fig. 6a, b). In addition, this spatial learning and memory impairment was also observed during the probe test, when the platform is removed on day 5 of the paradigm (Fig. 6c–g). Here (data expressed as mean ± SEM), untreated APP/PS1 mice crosses the area previously occupied by the platform fewer times (2.2 ± 0.5 times) than their wild type littermates (3.3 ± 0.4 times). Transgenic mice treated with ACR16 exhibited improved recall capacities, crossing the old platform location more times (3.8 ± 0.5 times) than untreated mice (*p* < 0.05; Student’s *T*-test) (Fig. 6e). These differences are not only noticeable when comparing the most representative records of each group (Fig. 6c), can also be observed by overlapping the performance of all the components of each group (Fig. 6d). These frequency graphs illustrate how the occupation of untreated APP/PS1 mice is lower than treated and nTg mice, observing a bigger predominance of blue in the platform region and most of the quadrants. We did not observe significant differences between the 3 groups during the probe test (when the platform was removed from the SW quadrant) regarding total distance traveled, total number of crossings between the 4 quadrants, or in the average swimming speed of the mice (Suppl. Fig. 4a). In addition, there was no preference in any of the groups for any quadrant visited during the probe test (Supp. Fig. 4b). However, regarding the percentage of time spent in each quadrant, we have observed differences within each group (Suppl. Fig. 4c). Whereas APP/PS1 mice treated with ACR16 have a similar pattern of time spent in each quadrant, particularly with regard to the NE and SW quadrants, this trait was not observed in APP/PS1 mice treated with saline. These results indicate that APP/PS1 mice treated with ACR16 spent more time in the SW quadrant in the former area of the platform and when this area was reached, and the platform was not encountered, the mice repeatedly returned to the NE quadrant to retrace their swimming path to find the missing platform. APP/PS1 mice treated with ACR16 mice made more crosses over the former areas of the platform (Fig. 6e) were relatively faster to reach the former area of the platform (Fig. 6f) and spent more time over the former area of the platform within the SW quadrant (Fig. 6g). All together, these behavioral observations indicate an improvement in spatial memory of APP/PS1 mice treated with ACR16.

### ACR16 Activates Akt in Hippocampus of APP/PS1 Mice

**Fig. 7:**
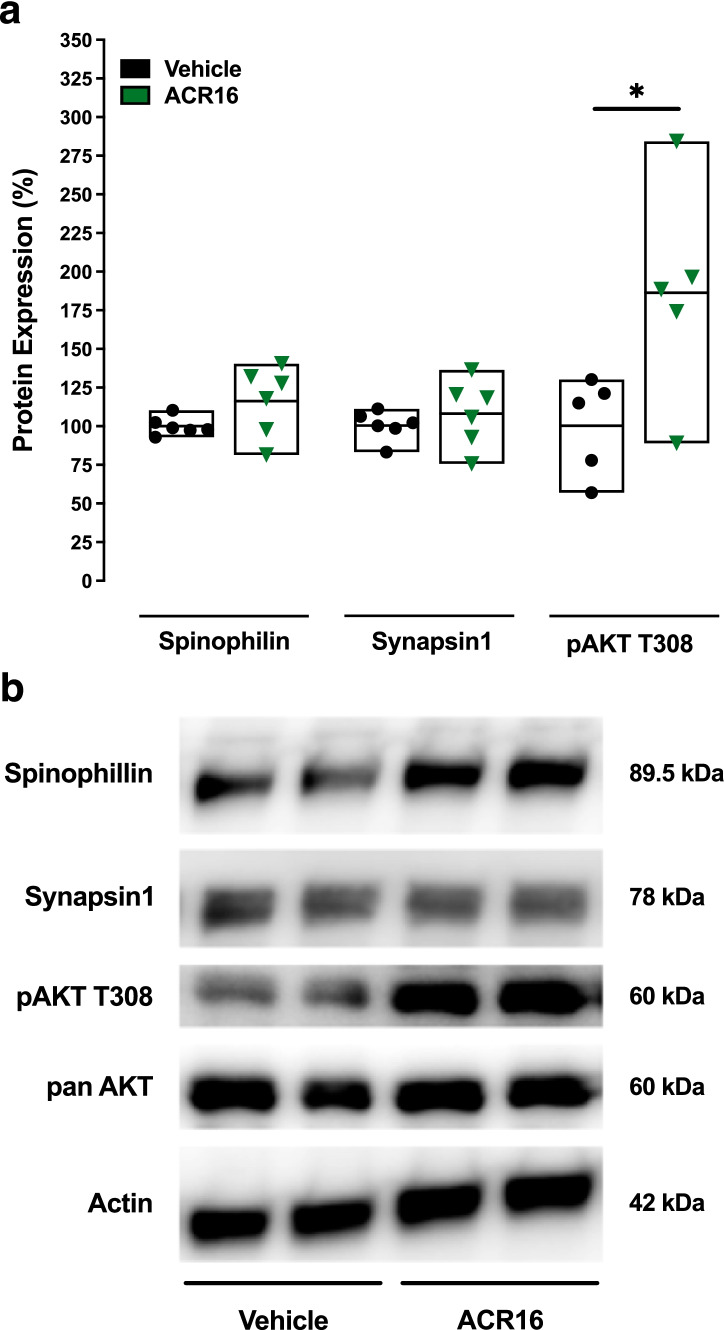
Systemic treatment with ACR16 increased the phosphorylation of AKT at T308 in hippocampus from APP/PS1 mice. Quantitative summary (**a**) and representative Western blot from 4 independent animals (**b**) evaluating spinophilin, synapsin1 protein expression (*n* = 6/group) and determining phosphorylated AKT levels (*n* = 5/group) in hippocampus of APP/PS1 mice treated with vehicle or 1.5 mg/kg ACR16. Quantitative densitometry of immunoreactive protein bands was normalized to those for actin (or pan-AKT in the case of pAKT) and then expressed as the percent change produced by ACR16 versus untreated controls [expressed as mean ± (Min–Max)]. Acute pridopidine treatment increased phosphorylated AKT at T308 (Student’s *t*-test, *(*p* < 0.05)). No changes were observed in expression of synapsin1 or spinophilin proteins

ACR16 exhibited more effective spinogenic effects in primary hippocampal neurons compared to PRE-084, in which ACR16 promoted the establishment of novel synapses, increasing the number of synapses per spine. This, together with the significant improvement in spatial recall capacities of Tg mice treated with ACR16, prompted the evaluation of synapsin1 and spinophilin gene and protein expression, as well as the status of PI3K-Akt pathway activation. Mice were sacrificed 3 h after completing the MWM paradigm, and both hippocampi were removed to quantify gene and protein expression. First, gene expression levels in hippocampus were evaluated using qPCR for synapsin1 (Syn1) and spinophilin (Ppp9r1b), among others. Although a slight increase was observed for spinophilin, no significant differences were observed (Suppl. Fig. 5). With regard to protein expression, no differences were observed in synapsin1 or spinophilin levels in mice treated with ACR16 (1.5 mg/kg/day, *i.p.*) (Fig. 7). However, ACR16 was found to significantly increase the phosphorylation of Akt at Thr308 in hippocampus [186 ± 31.0 (88.7–283.8), where data are expressed as mean ± SEM (Min–Max)], suggesting that ACR16-dependent effects observed in APP/PS1 mice are associated with activation of the PI3K-Akt pathway (Fig. 7).

## Discussion

Sigma 1 (σ1R) has been implicated in neurodegeneration, including excitotoxicity, calcium dysregulation, mitochondrial and endoplasmic reticulum dysfunction, inflammation, and astrogliosis [44]. A decreased density of σ1R has been observed in the brain of AD patients [5] and σ1R knockdown produces an increase in tau hyperphosphorylation, as well as mushroom spine destabilization reflecting AD pathology [45, 46]. Conversely, the increased σ1R expression, or its agonists-mediated activation, improves preexisting mechanisms of neuroprotection and neurorestoration demonstrated to slow disease progression [44]. Together, these findings have focused attention onto S1R as a promising therapeutic target for treatment of neurodegenerative and neuropsychiatric disorders [44]. Preclinical studies in several animal models suggest that S1R agonists are promising drugs for the treatment of cognitive dysfunction [44], suggesting a new, and hopeful, pathway in development of novel therapies for neurodegenerative diseases as Alzheimer’s disease.

Here, we report that two σ1R ligands, pridopidine (ACR16) and PRE-084, protect against NMDA-dependent excitotoxicity and increase neuronal survival in vitro (Fig. 1). Interestingly, our data indicated a concentration–response relationship of ACR16, whereas PRE-084 exhibited a similar effect at low or intermediate concentrations. In agreement with our results, σ1R involvement has previously been described in the modulation of calcium homeostasis and glutamate activity [44]. Moreover, previous findings have suggested a role of Aβ-peptide, generated in mitochondria-associated ER membranes [47], in mediating AD-related excitotoxicity [48] by its physical interaction with NMDAR [49] or by altering glutamate uptake and recycling mechanisms [50]. Excessive NMDAR activity would trigger a massive influx of calcium that would reach pathological levels, resulting in the neuronal death associated with AD [49–51]. These findings prompt us to consider future investigations to use σ1R ligands to revert Aβ42-dependent excitotoxic damage.

Previous studies reported a significant involvement of oxidative stress in aging and age-related neurodegenerative pathogenesis [36]. Specifically, oxidative stress occurs during early stages of AD and is detected in brain of mild cognitive impairment (MCI) patients [52, 53]. Increased levels of Aβ-peptide in AD generate further alterations in metal ion homeostasis and mitochondrial dysfunction that eventually led to the accumulation of ROS and the decline in antioxidant defense systems [50, 54, 55]. In our study, we report a neuroprotective effect of σ1R ligands in a model of oxidative stress in rat hippocampal cultured cells (Fig. 1 and Suppl. Fig. 3). In addition, this effect was found to be concentration dependent for ACR16. The reduction in cell death after both ACR16 and PRE-084 treatments (Fig. 1) was further corroborated by a reduction in the carbonylated protein content, a hallmark of oxidation status of proteins [54] (Fig. 1). Protein carbonylation is increased in AD-affected brain regions, including hippocampus, due to oxidative stress [54]. We found that 70 nM ACR16 but not 50 nM PRE-084 significantly reduced protein carbonylation around 42% (the decrease was 30% for PRE-084). Interestingly, Schreiner et al. [47] demonstrated that Aβ peptides are produced at MAMs, cellular microdomains where σ1Rs are principally located. Together, these outline the potential neuroprotective role for σ1R activation to prevent cellular damage produced by oxidative stress.

Dendritic spines are dynamic structures that provide the anatomical substrate for memory and learning processes [56, 57]. Moreover, spine number dramatically decreases with age and neuropathological diseases, such as AD [58]. Debilitating dementia associated with AD is a consequence of an early and progressive loss of synapses and dendritic spines [59]. In fact, the reduction in the number of hippocampal synapses is a pathological trait that has been detected in MCI individuals [60, 61] and AD mouse models [14, 62, 63]. Several lines of investigation suggest that synapse loss may result from the progressive accumulation of hyperphosphorylated tau protein [64] or/and Aβ-peptide [65]. In turn, some previous work points to deposits of aggregated Aβ, or amyloid plaques, alter intracellular calcium homeostasis, which produces neuritic dystrophy and dendritic spine loss [66]. In this study, we have documented the synaptogenic and spinogenic role played by both ACR16 and PRE-084 σ1R ligands (Figs. 2 and 3). Interestingly, recent data from primary cultured cells isolated from HD and/or AD mouse models indicate that ACR16 or PRE-084 modulates the release of calcium from ER, thereby regulating cytoplasmatic calcium levels [11, 67, 68]. It is plausible that a normalization of calcium homeostasis by ACR16 or PRE-084 at dendritic spines represents a mechanism to prevent synapse and spine loss and promote a stabilization of mature mushroom spines [37]. However, this interpretation overlooks the possibility that a decrease in synapse and spine loss might actually be a direct consequence of synaptogenesis and spinogenesis by σ1R ligands. To address this issue, we performed experiments in primary hippocampal cell cultures from Sprague–Dawley newborn rat pups to evaluate the effects of σ1R ligands, as opposed to primary cell cultures from a Tg animal model of AD. We established that both ACR16 and PRE-084 produce an increase in the number of synapses on dendrites (Fig. 2), and that ACR16 exhibited a greater synaptogenic effect than PRE-084, which resulted in a greater number of synapses per spine (Fig. 3c). Likewise, both σ1R ligands promoted the formation of new dendritic spines, but whereas PRE-084 is more spinogenic than ACR16 (Fig. 3a), showing a higher number of developing stubby synapses (Table 2), new spines from ACR16 exhibited a greater degree of maturation than PRE-084 (Fig. 3c) presenting a higher number of consolidated mushroom type spines (Table 2). We also observed that control cultures show a higher number of the mushroom spine type than treated cultures (Table 2). This difference may depend on the lower doses (70 nM for ACR16 and 50 nM for PRE-084, respectively) and treatment duration (48 h) used in our study. We have observed that both ACR16 and PRE-084 treatments generate more spines of the stubby type, but probably the dose and treatment time employed was not enough for the consolidation of mushroom spines. Further works employing different doses and longer treatment times with both ligands will give an answer to this possibility. Taken together, these results demonstrate that low concentrations of ACR16 and PRE-084 promote synaptogenesis and spinogenesis, whereas ACR16 exhibited a greater synaptogenic and spine maturation effect than PRE-084.

The protein kinases MAPK and PI3K modulate different physiological processes [69, 70]. In neurons, MAPK/ERK and PI3K/Akt/mTOR signaling pathways promote cell survival [71], play crucial roles in synaptic plasticity, learning, and memory processes, and also influence the morphogenesis/remodeling of dendritic spines [72, 73]. Interestingly, PI3K/Akt signaling controls synaptogenesis and spinogenesis both in vitro and in vivo in *Drosophila* flies and in mammals [20, 21, 74]. Moreover, a link between AD and dysfunctional MAPK/ERK and PI3K/Akt/mTOR signaling cascades is well documented [69, 75]. We evaluated these signaling pathways in context with the neuroprotective, synaptogenic, and spinogenic effects of ACR16 and PRE-084, showing how ACR16 (70 nM) and PRE-084 (50 nM) activated MAPK/ERK and PI3K/Akt/mTOR signaling cascades by increasing the phosphorylation of ERK1/2 and AKT, respectively, and confirming previous observations of σ1R ligands [37, 76]. In addition, both ACR16 and PRE-084 produced an increase in the expression of synapsin1 (Fig. 4) that was temporally preceded by increases in phosphorylation of ERK1/2 and AKT. These observations point toward the involvement of MAPK/ERK and PI3K/Akt/mTOR signaling pathways in the neuroprotective properties and synaptogenic capacity of σ1R agonists. A possible link between the increases in phosphorylated Akt and ERK 1/2 observed and increases in synapsin1 could be a PI3K/Akt/mTOR cross-activation by MAPK/ERK signaling via Ras-GTP [70]. A key element in this proposed mechanism could be glycogen synthase kinase 3 (GSK3). GSK3 is a serine/threonine protein kinase associated with AD that converges on both pathways [66]. GSK3 regulates multiple cellular processes in the brain [21] and both MAPK/ERK and PI3K/Akt/mTOR cascades modulate its activity [70]. Furthermore, it is established that GSK3 inhibition promotes synaptogenesis and spinogenesis [21, 74]. The role of GSK3 as a possible mechanism behind the synaptic and neuroprotective effects of σ1R ligands should be further explored.

Arc is an immediate early gene (IEG) that participates in neuronal plasticity [77] and its altered expression is related to AD pathophysiology and synapse loss [78]. In primary hippocampal cell cultures, we observed that ACR16, but not PRE-084, significantly increased Arc expression (Fig. 5). This increase together with the prolonged activation of MAPK/ERK and PI3K/Akt signaling pathways by ACR16 (Fig. 4a, b) may explain its greater synaptogenic and spinogenic effect (Figs. 2 and 3). The lack of increases in Arc expression by low concentrations of PRE-084 further highlights differences between these two σ1R ligands. Our observations are in line with those of Tadić et al. [79], where differences were reported between PRE-084 and SA4503, another σ1R agonist, despite belonging to the same family.

Deficits in working memory in APP/PS1 mice can be observed in the Morris water maze in aged mice [80]. These deficits in learning and memory are related to a sex-dimorphic overproduction of APP and Aβ [81] that results in neuronal loss and a reduction in the number of synapses in females [82]. Due to the stronger synaptogenic and spinogenic effect of ACR16 in primary neurons and its great potentiality on future clinical evaluation [5], we choose to examine whether ACR16 evokes pro-cognitive effects in 12-month-old APP/PS1 female mice. Based on our earlier work demonstrating the specifically of ACR16 at low doses at σ1R in vivo [9] and considering the results obtained from in vitro evaluation of ACR16, a low dose of ACR16 was also chosen. We have tested and found that a sub-chronic low-dose administration of ACR16 (1.5 mg/kg/day) significantly reduces working memory deficits in APP/PS1 mice (Fig. 6a, b). Improvement was observed during the acquisition phase and for memory recall during the probe test (Fig. 6e, f and Suppl. Fig. 4). Importantly, ACR16 was also found to significantly increase phosphorylated AKT compared to saline-treated APP/PS1 mice (Fig. 7a). However, contrary to what was observed in cell cultures, no significant changes were observed in protein expression of synapsin1 or spinophilin in the hippocampus of mice (Fig. 7), nor in gene expression of Arc (Supp. Fig. S5). The origin of these discrepancies is currently unknown but may be associated with the limitations of the techniques used in the current study. For example, Western blot analysis of total hippocampal lysates may not be sensitive enough to detect specific regional increases in spines and synapses. With this in mind, the procognitive effect of ACR16 in APP/PS1 mice may be a product of the combined synaptogenic and spinogenic properties observed in vitro on primary hippocampal neurons at 21-DIV yet increases in the expression of synapsin1 or spinophilin proteins were not observed in 12-month-old mice*.* Additional studies, with sub-chronic or chronic administration of pridopidine may be required to observe such changes in vivo using more advanced techniques. Beneficial properties of higher doses of ACR16 have been also reported in animal model of Huntington’s disease, Parkinson’s disease, and amyotrophic lateral sclerosis [11, 12, 83]. Differences in effective doses may reflect upon the animal model of disease that was studied.

In conclusion, we demonstrate neuroprotective and neurorestorative effects of sigma-1 ligands that result in new synapse and spine formation likely mediated by MAPK/ERK and PI3K/Akt signaling pathways in primary rat hippocampal cell cultures. Moreover, the present study confirms earlier reports of a neuroprotective role of sigma-1 agonists in AD mouse models [37]. These results are the first to demonstrate the efficacy of sub-chronic ACR16 improving spatial learning and memory in cognitively impaired AD mice. Taken together, our results underline the potential use of ACR16 as a pharmacological tool and put forward its future evaluation in AD and other dementias.

## Supplementary Information

Below is the link to the electronic supplementary material.

**Suppl. Fig. 1 Confocal microscopy image to illustrate the identification of neuronal versus glial cells.** Cell nuclei are labeled with DAPI (red) while only neurons have immunoreactivity to MAP2 (green). Yellow arrows indicate apoptotic or pyknotic nuclei from dying cells. (EPS 2305 kb)

**Suppl. Fig. 2 Protocol to discriminate viable cell nuclei from pyknotic nuclei or debris.** As an arbitrary criterion, we consider that nuclear condensation under 50 μm^2^ is indicative of cell death. Indeed, in cell nuclei above 50 μm^2^, we distinguished proper nucleoli and chromatin structures [see nuclei in the fluorescence confocal microscopy image (upper panel) and labelled in black (middle panel)]. In contrast, we observe cell nuclei less than 50 μm^2^ that are rounded and pyknotic. These can be observed in the optical image on the left and are labelled red on the right (lower panel). For this reason, after NMDA and H_2_O_2_ treatment, we quantified living cells as those cells with DAPI-labelled nuclei with an absolute area between 50 and 500 μm^2^, together with positive MAP2B labelling (green, upper panel). (EPS 19285 kb)

**Suppl. Fig. 3 S1R ligands did not influence neuronal survival or cell viability in the absence of NMDA or H**_**2**_**O**_**2**_ **and protect against neuronal death in a H**_**2**_O_**2**_**-dependent oxidative stress context.** Percent neuronal survival **(a)** and cell viability **(b) **normalized to control conditions for the two S1R agonists studied. Data are from cell cultures exposed for 24h with 500 nM PRE-084 or with 700 nM ACR16 (n=4 for each condition) and are calculated and expressed as described in Methods. Quantification of MAP2B-positive surviving neurons (number of neurons per mm^2^) in control, H2O2-treated (100 µM), H_2_O_2_+ACR16 (70 nM and 700 nM doses) and H_2_O_2_+PRE-084 (50 nM and 500 nM doses) conditions **(c)**. Statistical analysis was performed by 1-way ANOVA **(a,b)** and Student’s t-test **(c)** respectively. Significant statistical differences are displayed as *(p<0.05), **(p<0.01) and ***(p<0.001). (EPS 1317 kb)

**Suppl. Fig. 4 Probe test analysis in the MWM paradigm.** The analysis of total distance travelled, total number of crossings between the 4 quadrants, and average swimming speed of mice **(a)** during the probe test showed no differences between nTg, APP/PS1+saline and APP/PS1+ACR16 groups. The number of entries into each quadrant **(b)** was not different in any of the groups for any quadrant visited during the probe test. The analysis of the percentage of time spent in each quadrant **(c)** provided differences within each group. Data analysis for nTg (F (2.538, 22.84) = 5.65; **(p<0.01)), APP/PS1 +saline (F (1.636, 8.181) = 14.32; n.s.) and APP/PS1+ACR16 (F (1.650, 8.252) = 6,957; *(p<0.05)) included 1-way ANOVA followed by Tukey’s multiple comparisons test. (EPS 1556 kb)

**Suppl. Fig. 5 qRT-PCR quantification of transcript expression of selected genes in ACR16 treated and untreated 12-month-old APP/PS1 mice 4h after completing the MWM paradigm.** The percent change in mRNA levels between 1.5 mg/kg ACR16 treated mice (n=6) and vehicle treated mice (n=5) are provided. No differences were found. Statistical analysis was performed by unpaired Student's t-test and data are represented as mean ± S.E.M. (EPS 23380 kb)

Supplementary file6 (PDF 508 kb)

Supplementary file7 (PDF 517 kb)

Supplementary file8 (PDF 517 kb)

Supplementary file9 (PDF 517 kb)

Supplementary file10 (PDF 517 kb)

Supplementary file11 (PDF 1591 kb)

Supplementary file12 (PDF 1592 kb)
